# Persistence of HBV Vaccine’s Protection and Response to Hepatitis B Booster Immunization in 5- to 7-Year-Old Children in the Kohgiloyeh and Boyerahmad Province, Iran

**Published:** 2010-03-01

**Authors:** Behrouz Yazdanpanah, Mitra Safari, Shahrzad Yazdanpanah

**Affiliations:** 1 School of Paramedical, Yasouj University of Medical Sciences, Yasouj, Iran; 2 School of Nursing and Midwifery, Yasouj University of Medical Sciences, Yasouj, Iran; 3 School of Medicine, Yasouj University of Medical Sciences, Yasouj, Iran

**Keywords:** Hepatitis B Vaccine, Booster dose, Immune Protection

## Abstract

**Background and Aims:**

The duration of the protection of hepatitis B vaccination for infants and the necessity of a booster dose administration is unknown. The aim of the present study was to evaluate the persistence of seroprotection after hepatitis B virus (HBV) vaccination in order to determine the necessity of a single booster dose in 5- to 7-year-old children.

**Methods:**

This clinical trial study was conducted from 2004 to 2005.The study population included all children aged 5 to 7 years old in the Kohgiloyeh and Boyerahmad province who had been vaccinated starting at birth with hepatitis B vaccine. Among these children, 729 were selected via a multiple-stage sampling method. Parents gave their informed consent, and blood specimens (3 ml) were obtained from children. Hepatitis B surface antibody (HBsAb) and hepatitis B surface antigen (HBsAg) were determined by enzyme-linked immunosorbent assay (ELISA). Subjects with nonprotective titer levels (< 10 mIU/ml) received a booster does of the DNA recombinant vaccine. Four weeks after the administration of a booster dose, the antibody to HBsAg (anti-HBs) titer was measured. Data were analyzed using SPSS software, and analyses included chi-square, ANOVA, and independent-samples and paired-samples t-tests.

**Results:**

615 children (84.4%) had a protective antibody titer. The mean antibody titer was 230.5 ± 308.9 IU/ml, with a range of 10.6 to 1175 IU/ml. 15.6% of subjects had a nonprotective antibody titer, and the mean antibody titer was  4.97 ± 3.5 IU/ml. All subjects were HBsAg negative. No statistically significant differences were found by sex or by urban versus rural area of residence. The seroprotection rates significantly decreased by as the age of the children increased.Following the booster dose, 78.1 % of the children had a protective titer, and the mean titer significantly increased from 4.97 ± 3.5 at birth to 332.1 ± 402 IU/ml after the booster (P < 0.001).

**Conclusions:**

According to our results, the proper response of the immune system to a booster dose of HBV at 5 to 7 years of age reveals that immunologic memory is good after primary vaccination. Administration of booster dose does not appear to be necessary at these ages even though these children have a greater possibility of exposure to HBV in school age.

## Introduction

Hepatitis B virus (HBV) infects more than 350 million people worldwide. It is a leading cause of chronic hepatitis, cirrhosis, and hepatocellular carcinoma, and these sequels of chronic infection account for more than 1 million deaths annually [1].Immunization of infants against HBV has proven to be the most effective way to prevent infection [2].Approximately 3% of Iranian people are chronic carriers.However, the rate is different among provinces [3]. In Kohgiloyeh and Boierahmad province the incidence rate was 15.3 per 100,000,which is higher than the mean rate for the country as a whole (12.4 in 100000) [4]. The National Committee of Expanded Program of Immunization has approved the addition of hepatitis B vaccine to the previous immunization program, which has been in effect since 1993. The program ensures that all newborns receive all three doses of recombinant hepatitis B vaccine shortly after birth, one month later, and then at 6 months age.The standard HBV antibody (anti-HBV)vaccination elicits a protective antibody to hepatitis surface antigen (anti-HBs) levels in most people. However, the persistence of anti-HBs’s protective levels after vaccination and the effective duration of immunologic memory is not well understood [5]. The measurement of post vaccination serum level of anti-HBs is the only simple test available to predict the waning of protection and help plan for the administration of a booster dose [6].In unresponsive subjects a booster dose has been shown to be efficient, although the optimal method of management seems to depend on anti-HBs levels and has not been fully understood. The proper timing of booster dose administration is also not generally agreed upon [7].A number of studies in Iran have examined the long-term persistence of antibody after hepatitis B immunization at birth, and a few of these studies have assessed the challenges with administering a booster dose of the vaccine later in life. This study was conducted to evaluate the persistence of antibody after HBV vaccination and to assess the response to a booster dose in 5- to 7-year-old children.

## Materials and Methods

This research is a clinical trial performed from 2004 to 2005 in the Kohgiloyeh and Boyerahmad province of Iran. The study’s population included children aged 5 to 7 years old who were given a full three-dose hepatitis B vaccine as an infant (recombinant Engerix- B Biotec Havana, Cuba, 10µg). Households that were attended by public health centers were divided into 100 clusters based on the population served: This resulted in 46 urban and 54 rural clusters. From these clusters, 729 children were selected by a multiple-stage sampling method. All parents were requested to sign an informed consent form in which they agreed to participate in the study. After observing the children’s vaccination sheets and interviewing parents for collecting data, blood specimens (3 ml) were obtained from the children. All specimens were tested by an ELISA method (Radim kit- Tahghighgostar Co., Iran) for hepatitis B surface antibody (HBsAb) and hepatitis B surface antigen. Anti-HBs concentrations were measured and reported in IU/ml.The history of hepatitis B infection was noted in the parents’ reports. All children with an anti-HBs concentration ≥10 IU/ml were considered seroprotected and excluded from further analysis. Subjects with anti-HBs titer < 10 IU/ml received a booster dose of a recombinant vaccine intramuscularly in the deltoid muscle. Blood specimens were obtained from these children 4 weeks after the administration of the booster dose, and the anti-HBs titers were measured again with ELISA. The antibody titers after the booster vaccination were classified in three groups: 1) non-responders, who had anti-HBs levels <10 IU/ml; 2) responders with anti-HBs levels ≥10 IU/L and ≤ 100 IU/ml; and 3) high responders, who had anti-HBs levels > 100 IU/ml.Data analyses were performed using Chi-square, independent-samples t-test, paired-samples t-test, and ANOVA test. P < 0.05 was considered significant, and all P values were two sided.We assessed the effects of gender, age group, and residency on the development of anti-HBs ≥ 10 IU/ml and on the child’s response after the booster dose administration.The inclusion and exclusion criteria in this study were as follows. First, children who had received all three doses of the vaccine at infancy were included. Of this sample, children were excluded if they had a history of HBV infection, positive HBsAg, or a history of allergic reactions after receiving hepatitis B vaccine (used for the booster dose). These criteria were verified by reviewing children’s growth and vaccination sheets and by interviewing parents.

## Results

Seven hundred and twenty-nine children were enrolled in the study. Four hundred and twenty-five (58.3%) were males and 304 (41.7%) were female.Three hundred forty-eight (47.7%) of the children lived in urban areas, and 381 (52.3 %) lived in rural areas. One hundred fifty four (21.2%) were 5 years old, 257 (35.3%) were 6 years old, and 318 (43.5%) were 7 years old.The overall seropositivity rate for anti-HBs (≥10I U/ml) was 84.4% (615 children), and in 39.1% (285 children) of these children the antibody concentration level was higher than 100 IU/ml. One hundred fourteen (15.6%) children had anti-HBs concentrations < 10 IU/ml (non-seroprotective). All the children tested negative for HBsAg and had no history of HBV infection or clinical evidence of infection or disease.The mean anti-HBs concentration in seropositive children was 230.5 ± 308.9 IU/ml, ranging from 10.6 to 1175. In children without a seroprotective titer (no-seroprotective children), the mean antibody concentration was 4.97 ± 3.5. The seroprotection rate and its relationship with the demographic variables such as sex, age, and residential area is shown in Table 1.The females had a significantly higher mean anti-HBs titer than males (231.5 ± 320.1 versus 169.3 ± 273.6, P = 0.006).The mean anti-HBs titers were 232.5 ± 316.7,205.4 ± 305.1, and 169 ± 274.2 in 5-, 6-, and 7-yearold children, respectively. This differences were not statistically significant (P = 0.07).Table 2 shows the anti-HBs concentration in nonseroprotective children after receiving the booster dose. As shown in Table 2, 89 out of the 114 nonseroprotective children (78.1%) children responded with anti-HBs level ≥ 10 IU/ml after the booster dose. The mean anti-HBs concentration in children who received the booster dose was 332.1 ± 402 IU/ml after the booster, which was approximately 67 times higher than the mean antibody concentration before the booster. This difference was statistically significant (P < 0.05). There were no significant differences in seroprotectivity after the booster dose by sex, age group, or area of residence.

**Table 1:** Anti-HBs concentration according to the demographic characteristics of vaccinated children.

**Characteristic**		**Anti-HBs Concentration**	**≥10IU/ml No. (%)**	**<10 IU/ml No. ( %)**	**Total No. (%)**	**P-value**
Sex	Male		356(83.8)	15(16.2)	425(100)	
	Female		259(85.2)	39(14.8)	304(100)	N.S
Residency	Urban		294(84.5)	54(15.5)	348(100)	
	Rural		321(84.3)	60((15.7)	381(100)	N.S
Age(years)	Five		139(90.3)	15(9.7)	154(100)	
	Six		218(84.8)	39(15.2)	257(100)	0.04
	Seven		258(81.8)	60(18.9)	318(100)	

**Table 2:** The anti-HBs concentration in non-seroprotective children after receiving the booster dose.

**Antibody concentration**	**Number of subjects**	**Percent**
<10 IU/ml	25	21.9
≥10 IU/ml and ≤ 100IU/ml	28	24.6
> 100IU/ml	61	53.5
Total	114	100

## Discussion

The results of this study indicate that the majority of the participants (84.4%) who had been immunized with recombinant HBV vaccine in their first years of life had a protective concentration of antibody at 5 to 7 years of age, and 285 (39.1%)of these children had high anti body levels. Our results are similar to those by Salehi et al. [8], who reported that, 6 years after vaccination, 81% of Iranian children had protective antibody levels and 29.2% had high antibody levels. Mahyar reported an 87.5% seroprotection rate at 6 years old and Behjati et al. reported a 69% rate at 7 years old in Iranian children after primary vaccination [9][10]. Garcialiop et al. reported a 70.6% seroprotection rate in Spanish children 6-7 years after vaccination [11].Most studies have found that there were no gender differences in the immunological response to HBV vaccine [12][13][14]. Similarly, in our study the seroprotection rates of girls and boys were not statistically different. However, mean antibody titer in girls was significantly higher than the titer in boys; this suggests that females are stronger responders.This finding is similar to of the results in Gold et al.and Salehi et al. [8][15].In this study the seroprotection rate and mean antibody titer were found to decrease with age and had significant associations with the age of the children. On the other hand, child’s age negatively influenced anti-HBs level. This result corroborates findings from previous studies that indicate that anti-HBs concentrations decline over time [16][17][18][19][20][21][22].In our study the majority of subjects (78.1%)responded to the booster dose, and more than half of them had a high antibody level (>100 IU/ml).Other studies on children have shown similar results [16][18][23].Immunologic memory was confirmed by the high response to the administration of the booster dose in the children with an anti-HBs concentration < 10 IU/ml 5-7 years after the original vaccination series.Exposure to the booster antigen in these children stimulated the antibody concentration to exceed 100 IU/ml in more than half of all cases. The statistically significant difference between the mean antibody concentration before and after the booster dose shows the vigorous response of the immune system to the booster and suggests that the immunologic memory is good.Similarly, Gonzales et al.’s follow-up study of HBV-vaccinated children showed that the mean antibody titer increased significantly from 34 to 2,985 IU/L after a booster in 7-year-old children (P< 0. 001) [24].Evaluating the response to a booster dose of the HBV vaccine in subjects with history of vaccination at least five years ago resulted in approving the presence of memory response to the HBs antigen as reported in several studies [24][25][26][27][28][29][30][31][32].We found no cases of HBsAg reactivity in our study subjects, including those who were no longer anti-HBs seropositive. The reason for this data may be indicative of a lack of contact with HBV or induced immunological memory due to their primary immunization of this age group. There is no medical evidence, however, to show that healthy vaccinated children lose their immunity simply because the anti-HBs level drops below 10 IU/ml [33]. In two studies carried out on Chinese adolescents 13 to 15 years after they had been vaccinated at birth, the results showed that fewer than 2% of the adolescents were HBsAg seropositive, despite a seroprotectivity rate of less than 50% in these studies [34][35].In conclusion, our study on 5- to 7-year-old children who were vaccinated early in life showed that an effective seroprotection level persisted in 84.4% of subjects. Given this result, that the response to the booster dose in 78.1% of children did not result in a seroprotective level, and the absence of HBsAg in all subjects, we conclude that a routine administration of an HBV booster dose is not necessary before 7 years of age. Further follow-up studies on older children and adolescents are needed to verify the persistence of seroprotectivty, immunologic memory, and the necessity of booster dose administration at these ages. In the present study, about 22% of children with a non-protective level of anti-HBs did not respond to the booster dose. More studies are needed to evaluate the necessity of extra dose of booster in these children.
